# Nationwide implementation of integrated community case management of childhood illness in Rwanda

**DOI:** 10.9745/GHSP-D-14-00080

**Published:** 2014-08-05

**Authors:** Catherine Mugeni, Adam C Levine, Richard M Munyaneza, Epiphanie Mulindahabi, Hannah C Cockrell, Justin Glavis-Bloom, Cameron T Nutt, Claire M Wagner, Erick Gaju, Alphonse Rukundo, Jean Pierre Habimana, Corine Karema, Fidele Ngabo, Agnes Binagwaho

**Affiliations:** a Rwanda Ministry of Health, Kigali, Rwanda; b The Warren Alpert Medical School of Brown University, Providence, RI, USA; c Brown University, Watson Institute for International Studies, Development Studies Program, Providence, RI, USA; d Dartmouth Center for Health Care Delivery Science, Hanover, NH, USA; e Global Health Delivery Partnership, Boston, MA, USA; f Malaria and other Parasitic Diseases Division, Rwanda Biomedical Center, Kigali, Rwanda; g Harvard Medical School, Department of Global Health and Social Medicine, Boston, MA, USA; h Dartmouth College, Geisel School of Medicine, Hanover, NH, USA; * Co-first authors

## Abstract

**Background:**

Between 2008 and 2011, Rwanda introduced integrated community case management (iCCM) of childhood illness nationwide. Community health workers in each of Rwanda's nearly 15,000 villages were trained in iCCM and equipped for empirical diagnosis and treatment of pneumonia, diarrhea, and malaria; for malnutrition surveillance; and for comprehensive reporting and referral services.

**Methods:**

We used data from the Rwanda health management information system (HMIS) to calculate monthly all-cause under-5 mortality rates, health facility use rates, and community-based treatment rates for childhood illness in each district. We then compared a 3-month baseline period prior to iCCM implementation with a seasonally matched comparison period 1 year after iCCM implementation. Finally, we compared the actual changes in all-cause child mortality and health facility use over this time period with the changes that would have been expected based on baseline trends in Rwanda.

**Results:**

The number of children receiving community-based treatment for diarrhea and pneumonia increased significantly in the 1-year period after iCCM implementation, from 0.83 cases/1,000 child-months to 3.80 cases/1,000 child-months (*P* = .01) and 0.25 cases/1,000 child-months to 5.28 cases/1,000 child-months (*P*<.001), respectively. On average, total under-5 mortality rates declined significantly by 38% (*P*<.001), and health facility use declined significantly by 15% (*P* = .006). These decreases were significantly greater than would have been expected based on baseline trends.

**Conclusions:**

This is the first study to demonstrate decreases in both child mortality and health facility use after implementing iCCM of childhood illness at a national level. While our study design does not allow for direct attribution of these changes to implementation of iCCM, these results are in line with those of prior studies conducted at the sub-national level in other low-income countries.

## Abstract (precis)

Between 2008 and 2011, Rwanda introduced iCCM of childhood illness nationwide. One year after iCCM rollout, community-based treatment for diarrhea and pneumonia had increased significantly, and under-5 mortality and overall health facility use had declined significantly.

## INTRODUCTION

In the mid-1990s, the World Health Organization (WHO) and the United Nations Children's Fund (UNICEF) launched an Integrated Management of Childhood Illness (IMCI) strategy to reduce child deaths from pneumonia, diarrhea, measles, malaria, and malnutrition.^1^ The strategy focused on improving case management skills of health care providers, overall health systems, and family and community health practices.

By 2005, IMCI had been rolled out in 100 countries. An evaluation conducted in a subset of these countries highlighted both the successes and limitations of the IMCI strategy and stressed the importance of community-based case management to further reduce under-5 mortality.^2^ A joint statement by WHO and UNICEF acknowledged that, by providing community-based case management of childhood illnesses, trained community health workers (CHWs) could improve child survival rates.^3^

Community-based case management of childhood illnesses could improve child survival rates.

Despite major reductions in under-5 mortality over the past 2 decades, pneumonia, diarrhea, and malaria still cause more than 2.7 million child deaths each year.^4^ WHO and UNICEF estimate that timely diagnosis and provision of basic curative services for these diseases could reduce pneumonia deaths by 70%, diarrhea deaths by 70%–90%, and malaria deaths by 40%–60%.^5^ The Lancet Diarrhoea and Pneumonia Interventions Study Group recently indicated that impact evaluation of community case management of diarrhea and pneumonia in resource-limited settings was an urgent research priority.^6^ While several published studies have investigated the quality and effectiveness of care provided by local-level CHW programs, fewer studies have documented public-sector experiences in implementing integrated community case management (iCCM) for childhood illness on a national scale, and no prior studies have evaluated the impact of iCCM on both child mortality and health facility use at the national level.

By 2011, iCCM had been rolled out to all 30 districts in Rwanda. This article summarizes the iCCM implementation experience in Rwanda and then uses existing data sources to examine changes in child mortality and health facility use in the 1-year period after iCCM implementation in each district. In addition, we also report on changes in the number of children receiving community-based treatment for pneumonia, diarrhea, and malaria during this time period.

## ICCM IMPLEMENTATION IN RWANDA

After the 1994 genocide in Rwanda, community-based health activities were introduced, although it took another 10 years before a national iCCM policy was developed. Between 2003 and 2004, in response to persistently low antimalarial treatment rates for children under 5, Rwanda's Integrated National Malaria Control Program (INMCP) began drafting a strategic plan to introduce home-based management for malaria in 6 pilot districts and, in collaboration with the WHO Regional Office for Africa (AFRO), developed implementation guidelines for home-based management of malaria. The program was modeled after programs in Kenya and Uganda, using trained community volunteers to treat children with fever with prepackaged antimalarial drugs for presumed malaria.

In 2008, the Rwandan Ministry of Health (MOH) expanded this program, in partnership with a consortium of international nongovernmental organizations (NGOs) and with the support of the President's Malaria Initiative (PMI), the United States Agency for International Development (USAID), and the Global Fund to Fight AIDS, Tuberculosis & Malaria (the Global Fund). This Expanded Impact Program, or *Kabeho Mwana*, trained and equipped CHWs to provide community-based treatment for uncomplicated cases of diarrhea and pneumonia, in addition to malaria, in specific intervention areas. An early evaluation of *Kabeho Mwana* revealed that, in the 6 intervention areas, the proportion of children with diarrhea who received oral rehydration solution (ORS) increased from 19% to 33%, and that antibiotic use for pediatric pneumonia rose from 13% to 54%.^7^

**Figure 1.**
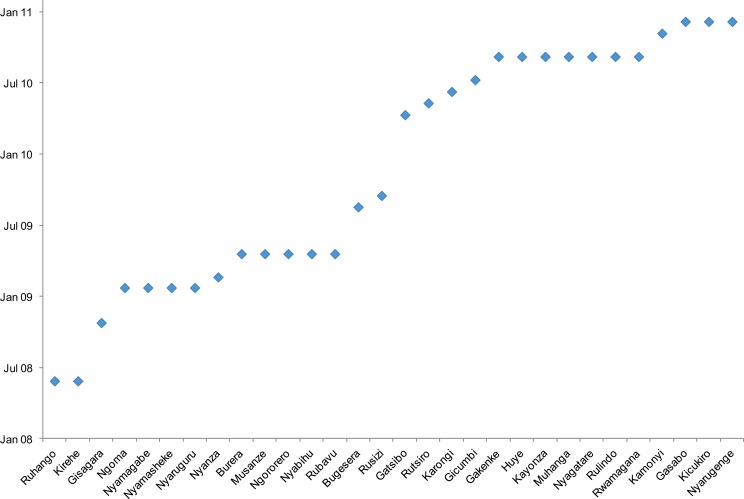
Date of Implementation of Integrated Community Case Management by District, Rwanda

Based on these data and the success of iCCM programs in other low-income countries, the MOH rolled out iCCM in all 30 Rwandan districts between June 2008 and January 2011 (Figure 1). During this implementation period, each of Rwanda's 14,837 villages elected 2 community members, 1 man and 1 woman, to provide comprehensive primary health care services—a ratio of approximately 1 CHW for every 50 children under 5 in the population. The majority of these CHWs are between the ages of 30 and 49, have completed primary school, and are employed as agricultural workers.^8^

By 2011, iCCM of childhood illnesses had been scaled up nationwide in Rwanda.

### CHW Training and Activities

Community health workers provide a variety of health promotion, diagnostic, curative, referral, and reporting services for their villages (Box). All CHWs have participated in a 5-day, 31-hour training course on iCCM at their closest health center. The course, taught by nurses in charge of community health activities for their sector, is based on *Community Case Management Essentials: Treating Common Childhood Illnesses in the Community*.^9^ The Rwanda MOH, with expert advice from WHO and UNICEF, adapted this guide to create locally acceptable treatment algorithms, which CHWs use to calculate age-specific doses of ORS and zinc for diarrhea; artemether-lumefantrine for malaria; and amoxicillin for pneumonia. The adapted training module also teaches CHWs to use malaria rapid diagnostic tests and respiratory rate timers to diagnose malaria and pneumonia, respectively.^10^

**BOX. iCCM Community Health Worker Activities in Rwanda**

**Health Promotion**

- Community sensitization about immunization, awareness of common diseases, hygiene and sanitation, birth spacing, family planning, and breastfeeding
- Growth monitoring and malnutrition surveillance

**Diagnostic and Curative Services**

- Diagnosis of malaria with rapid diagnostic tests
- Treatment of confirmed malaria with artemether-lumefantrine
- Treatment of acute respiratory infections with amoxicillin
- Treatment of diarrhea with oral rehydration solution and zinc

**Referral and Reporting Services**

- Equipped with cell phone to alert local health center and district hospital of emergencies and to call ambulances
- Report new births and deaths via text message to local health center and Ministry of Health
- Refer children with moderate and severe acute malnutrition, severe pneumonia, severe malaria, and severe dehydration to local health center or district hospital

### Quality Assurance

The nurse in charge of community health for each sector or a cell supervisor conducts quarterly site visits to evaluate the iCCM diagnostic and curative services provided by CHWs. The cell supervisor is a trained CHW appointed to provide iCCM program oversight in a catchment area comprising 5 or 6 villages.^11^ Specifically, the cell supervisor is responsible for compiling the CHWs' iCCM registers and verifying that the reported data are both complete and correct. Data are considered complete if the CHW has recorded the symptoms, disease classification, and prescribed treatment for each child included in the iCCM registry. For registry data to be considered correct, the total number of sick child visits must equal the sum of children treated and children referred.

The nurse in charge of community health organizes quarterly 2-day refresher trainings in conjunction with site visits. These training sessions provide CHWs an opportunity to ask questions about the iCCM package, treatment protocols, and data collection tools, as well as for the nurse to introduce changes to national iCCM treatment policy. For example, the MOH recently rolled out the *m'Ubuzima* program, which collects iCCM data through an interactive voice response program; the nurses have been tasked with teaching the CHWs how to use the new software on their government-issued cell phones. The MOH aims to have the *m'Ubuzima* program, in combination with RapidSMS reports, replace paper iCCM registers. This initiative is expected to increase the number of CHWs who complete monthly reports and improve the accuracy of those reports by eliminating human error in data aggregation.

### Financing

The CHWs are further held accountable for the quality of care they provide through a performance-based financing scheme. Community health workers are organized into cooperatives, which meet monthly at the health center in each sector. The MOH disburses funds to these cooperatives once per quarter on the basis of key health indicators, including number of households using insecticide-treated bed nets, appropriate management of diarrhea-related dehydration, and accurate data reporting in iCCM registers. The majority (70%) of performance-based financing grants are invested into income-generating projects chosen by cooperative vote. The remaining 30% is paid directly to CHWs as cash bonuses equivalent to approximately US$0.73 per month per CHW. The MOH has contracted with a local organization, Square Entrepreneurship Development Consult, to develop the business planning and financial management capacities of cooperatives as grants for performance-based financing from the Global Fund are phased out over time.

To encourage good performance, the MOH disburses funds through cooperatives to CHWs who have met key health indicators.

### Supply Chain

The Medical Procurement and Distribution Division (MPDD) of the MOH purchases medical supplies for the national health care system from both national and international pharmaceutical manufacturers. Purchased drugs and medical supplies are stored in district pharmacy warehouses until distribution to district hospitals and health centers. Medicines were previously available only at the district hospital; now all drugs earmarked for community distribution are sent to the health center. Cell coordinators obtain medicines from the health center on an as-needed basis and then supply the CHWs in their catchment area. This supply chain was developed to reduce the frequency of community-level stock-outs, which originally posed challenges for the home-based management for malaria program. The MOH has made additional efforts to improve supply chain management by partnering with John Snow, Inc., to develop a tool to more accurately forecast community-level demand for medical supplies.

### Policy and Partnership

In addition to monitoring iCCM indicators and managing performance-based financing grants, the MOH, through the Ministry's Community Health Desk (CHD) and Malaria and other Parasitic Diseases Division, also oversees iCCM protocol revision and develops CHW training tools. A Technical Working Group, which includes many of the same organizations that were involved in the development and implementation of the original *Kabeho Mwana* program, assists the MOH in these tasks. Overall funding for the iCCM program has come from the Government of Rwanda, the Global Fund, PMI, USAID, and the Canadian International Development Agency (CIDA).^10^ In addition, several NGO partners—Partners in Health, Concern Worldwide, and the International Rescue Committee—have played significant roles in Rwandan community-based health initiatives by providing technical, financial, oversight, and quality assurance support in individual districts.

## METHODS

### Data Sources

The primary data for this study were obtained from the Rwanda health management information system (HMIS) database, which collects data at both the community and health facility levels. The **community-based data** were derived from monthly CHW reports, which include the number of non-health facility child deaths in each village (referred to as community deaths) and the number of children treated for pneumonia, diarrhea, and malaria by CHWs (referred to as community-based treatment). These CHW reports are transferred to a cell supervisor, who regularly audits CHW data collection, as described earlier. Data officers at each health center then aggregate the data from all cell supervisors in their catchment area and enter them into the HMIS database.

The **health facility data** reported in the HMIS database include monthly counts of health center visits, district hospital admissions, and health facility deaths for every district, stratified by age group. To estimate total public-sector health facility use from these data, we summed the total number of health center visits and district hospital admissions for children under 5 for all causes. We derived health facility mortality by adding available health center mortality and district hospital mortality figures for all causes.

Finally, we combined available under-5 community mortality data with our under-5 health facility mortality data to calculate the all-cause under-5 mortality rates for each district.

### Population-Based Rates

We calculated population-based rates by dividing the crude HMIS data by district population size, derived from data published by the National Institute of Statistics of Rwanda (NISR). The NISR uses national census data to estimate annual population growth rates for each district based on changes in district population between the 2000 and 2010 national censuses. We used these estimates to determine district populations for each year of our study period. Based on NISR data collected for the 2010 Demographic and Health Survey (DHS), we assumed that 16.2% of the population was under the age of 5. We divided monthly district totals for community-based treatment, under-5 mortality, and health facility use by the estimated under-5 district population and multiplied by 1,000 to generate rates per 1,000 children under 5 in the population.

### Seasonal Matching

To measure the impact of iCCM on child health in Rwanda, we compared monthly averages for indicators of interest before and after iCCM implementation. Because iCCM was implemented at different times in different districts of Rwanda, and since child morbidity is seasonally variable, we compared a 3-month, pre-iCCM baseline period for each district to a seasonally matched 3-month comparison period 1 year after iCCM implementation. For each district, we calculated monthly rates for our indicators in both the baseline and comparison periods, averaging the monthly values within each period. If data were missing from all 3 months of a given period, we excluded that district from our analysis. We also performed a more conservative analysis by eliminating districts if even 1 month of data was missing from either the baseline or comparison period. For each indicator, we specified the number of districts providing complete data for analysis.

**Figure 2.**
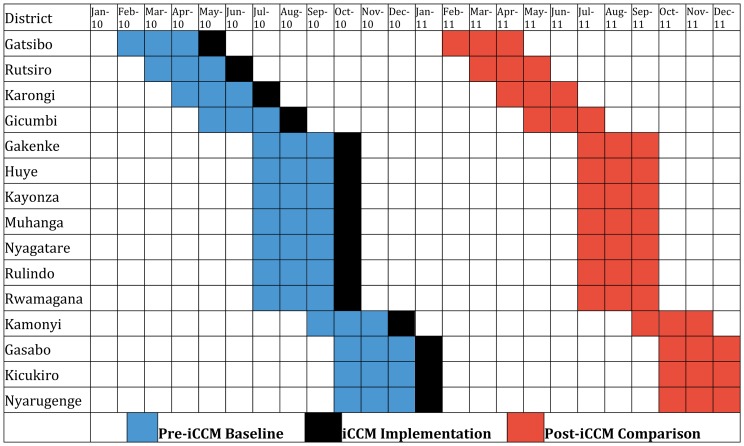
Seasonal Average Schematic Applied to Community Mortality Data, Rwanda Abbreviation: iCCM, integrated community case management.

Strict quality control mechanisms for data collected by CHWs were put in place beginning in late 2009. Therefore, reliable baseline data on the number of child deaths occurring in the community and the number of children treated for pneumonia, diarrhea, and malaria in the community are not available for most districts that implemented iCCM before 2010. Figure 2 illustrates the timing of the baseline and comparison periods we applied to the 15 districts for which we have complete community mortality data. Similar baseline and comparison periods were applied to those districts for which we have complete community-based treatment data, health facility use data, and health facility mortality data to calculate pre-iCCM and post-iCCM averages for each district for these additional indicators.

### Comparison of Actual Versus Expected Trends

To compare actual versus expected trends, we determined trends in total under-5 mortality and in health facility use in Rwanda prior to iCCM implementation. We then projected these trends to each individual district to determine the expected decrease in total under-5 mortality and health facility use during the 1-year period after iCCM implementation, assuming constant baseline trends. Next, we compared the actual changes to the expected changes for each district to determine whether the actual decreases in mortality and health facility use were greater than would have been expected due to baseline trends.

For total mortality, we used the UN Inter-agency Group for Childhood Mortality Estimation (CME Info) database to establish a baseline trend for under-5 mortality in Rwanda during the decade prior to iCCM implementation.^12^ To determine a baseline trend for health facility use, we used available data from the HMIS database, which began aggregating national health facility data in 2008. For each district where data were available, we calculated the annual percentage change in health facility use during the 1-year period prior to iCCM implementation and used this value to approximate the baseline percentage change in health facility use.

### Statistical Analysis

We used the Wilcoxon signed-rank test, which does not require data to be normally distributed, to compare community-based treatment, under-5 mortality, and health facility use rates for pre-iCCM baseline and post-iCCM comparison periods in each district. For comparison of actual and expected time trends in under-5 mortality and health facility use data, we again used the Wilcoxon signed-rank test to compare the actual change in each variable to the expected change in each variable. We considered alpha < .05 to be significant in all cases. All analyses were performed using SPSS version 20 (IBM, New York).

## RESULTS

### Treatment for Childhood Illness

Data from 21 districts revealed that the number of children who received community-based treatment for diarrhea and pneumonia increased significantly with iCCM implementation (Table 1). The average number of children with diarrhea who received treatment from a CHW rose from 0.83 cases/1,000 child-months at baseline to 3.80 cases/1,000 child-months during the comparison period, an average increase of 2.97 cases/1,000 child-months (95% confidence interval [CI] = 0.97–4.97; *P* = .01). The mean number of children who were treated for pneumonia increased from 0.25 cases/1,000 child-months to 5.28 cases/1,000 child-months, an average increase of 5.03 cases/1,000 child-months (95% CI = 3.06–7.00; *P*<.001).

**Table 1.** Treatment of Childhood Illnesses by Community Health Workers Before and After iCCM Implementation, Rwanda, 2010–2011, N = 21 Districts

**Illness**	**Baseline Period**	**Comparison Period**	**Difference (95% CI)**	***P* Value (2-tailed)**
Pneumonia	0.25	5.28	5.03 (3.06, 7.00)	< .001
Diarrhea	0.83	3.80	2.97 (0.97, 4.97)	.01
Malaria	19.14	7.27	−11.87 (−21.92, −1.83)	.03

Treatment data are reported as number per 1,000 child-months.

Community-based treatment of children for diarrhea and pneumonia increased significantly with iCCM implementation, while treatment for malaria decreased.

In contrast, the average number of children who were treated for malaria by a CHW decreased from 19.14 cases/1,000 child-months to 7.27 cases/1,000 child-months, representing a decline of 11.87 cases/1,000 child-months (95% CI = 1.83–21.92; *P* = .03). This decrease may be attributed to the introduction of rapid diagnostic tests for malaria as part of the iCCM treatment algorithm; with diagnostic testing, treatment is limited to children with positive rapid tests, as opposed to all children with fever.

### Under-5 Mortality

Baseline and comparison data for under-5 mortality were available in 15 of 30 districts. In these 15 districts, the community under-5 mortality rate declined by 47% during the year after iCCM implementation, from 0.38 deaths/1,000 child-months to 0.20 deaths/1,000 child-months (Table 2). This represents an average decline of 0.18 deaths/1,000 child-months (95% CI = 0.11–0.24; *P*<.001). By comparison, health facility under-5 mortality did not change significantly in the 28 districts for which baseline data were available (*P* = .41) (Table 2).

**Table 2.** Under-5 Mortality and Health Facility Use Before and After iCCM Implementation, Rwanda

**Indicator (No. of Districts)**	**Baseline Period**	**Comparison Period**	**Difference (95% CI)**	***P* Value (2-tailed)**
Community mortality (15)	0.38	0.20	0.18 (0.11, 0.24)	< .001
Health facility mortality (28)	0.09	0.10	−0.02 (−0.05, 0.01)	.41
Total mortality (15)	0.48	0.30	0.19 (0.10, 0.27)	<.001
Health center use (30)	2.48	2.00	0.48 (0.13, 0.82)	.009
District hospital use (28)	2.17	1.91	0.26 (−0.08, 0.59)	.10
Total health facility use (28)	4.57	3.88	0.69 (0.15, 1.23)	.006

All data are reported as number per 1,000 child-months. Because of rounding, differences may not appear exact.

Community under-5 mortality declined significantly with iCCM implementation, while health facility under-5 mortality did not change significantly.

**Figure 3.**
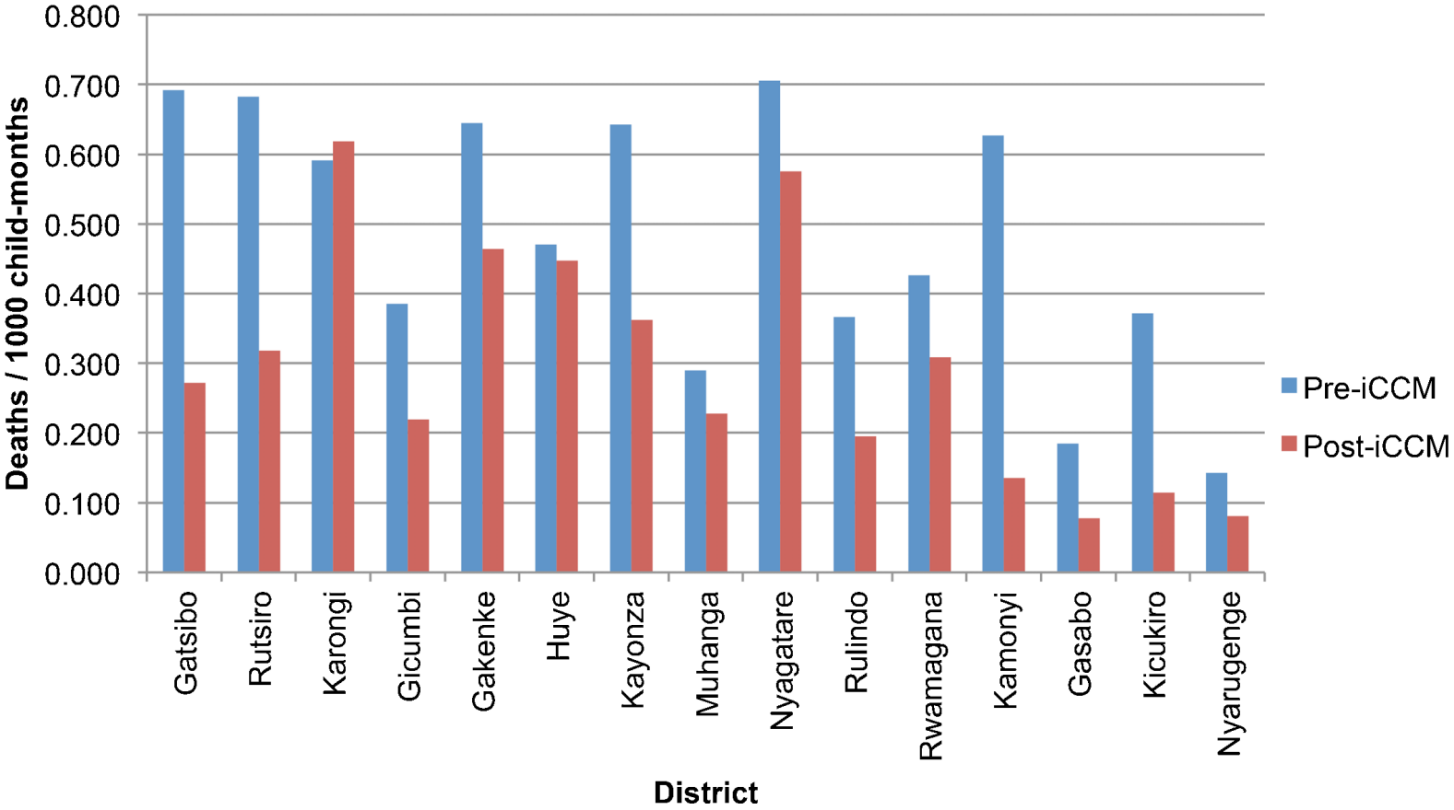
Total Under-5 Mortality by District, Rwanda Abbreviation: iCCM, integrated community case management.

Despite the lack of change in health facility mortality, total mortality did decrease significantly after iCCM introduction, since nearly three-quarters of child deaths in Rwanda occur in the community; in the 15 districts with both baseline and comparison data, total all-cause, under-5 monthly mortality rates declined by 38% during the 1-year period after iCCM rollout, from 0.48 deaths/1,000 child-months to 0.30 deaths/1,000 child-months, for an average decline of 0.19 deaths/1,000 child-months (95% CI = 0.10–0.27; *P*<.001). Total mortality declined in all districts except Karongi (Figure 3).

### Health Facility Use

Data from all 30 districts show that health center visits decreased from an average of 2.48 visits/1,000 child-months to 2.00 visits/1,000 child-months, representing a decline of 0.48 visits/1,000 child-months (95% CI = 0.13–0.82; *P* = .009), or a 19% decrease in health center use. District hospital admissions also declined during the study period, from 2.17 admissions/1,000 child-months to 1.91 admissions/1000 child-months in the 28 districts for which data were available, although the change was not statistically significant (95% CI = −0.08–0.59; *P* = .10).

**Figure 4.**
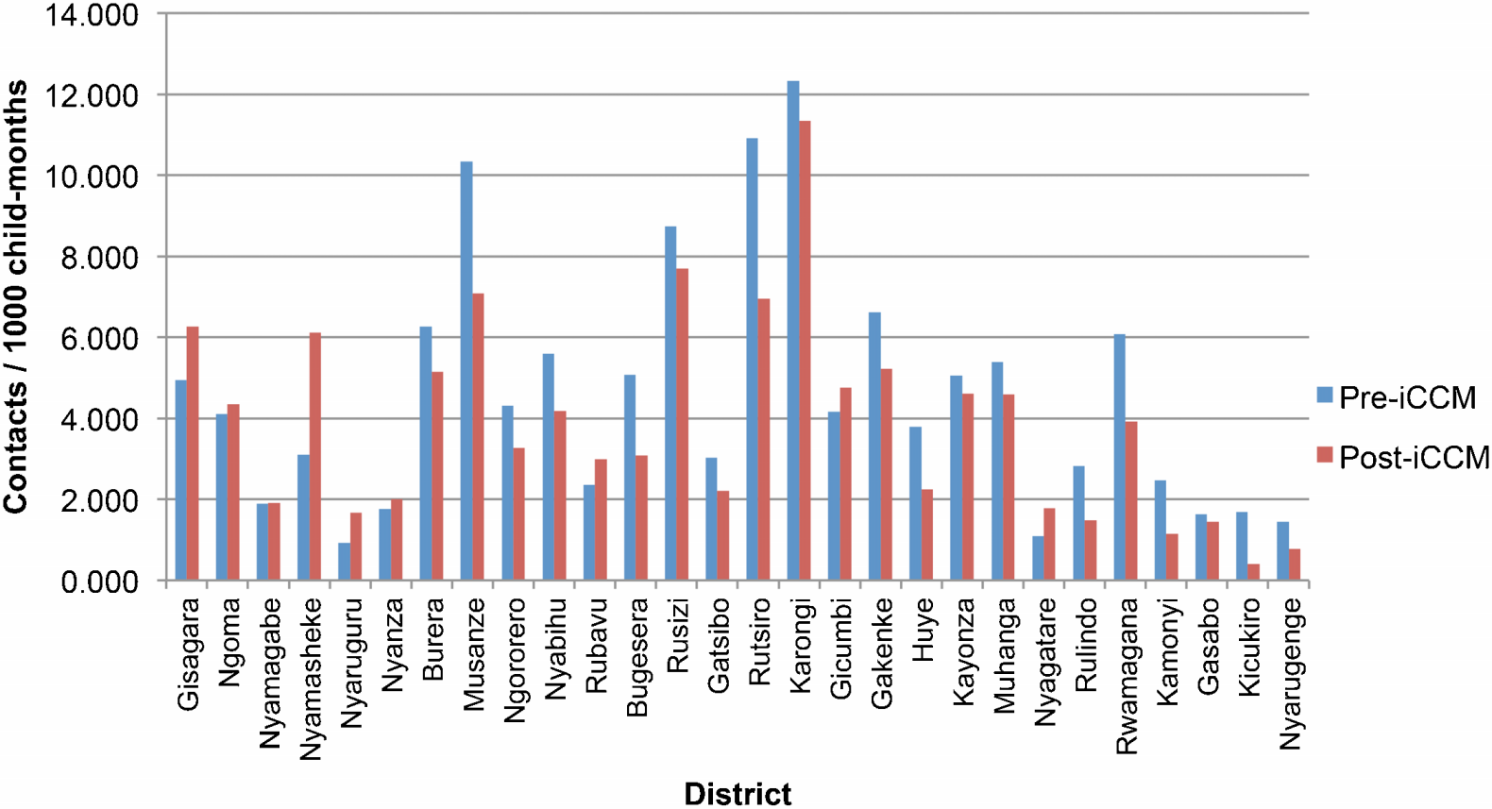
Total Under-5 Health Facility Use by District, Rwanda Abbreviation: iCCM, integrated community case management.

Finally, total health facility use for children under 5 for all causes declined significantly during the 1-year period after iCCM implementation in the 28 districts for which data were available, from 4.57 contacts/1,000 child-months to 3.88 contacts/1,000 child-months. This represents a decrease of 0.69 contacts/1,000 child-months (95% CI = 0.15–1.23; *P* = .006), representing a 15% decline. Total health facility use declined in 20 of the 28 districts in which data were available (Figure 4).

**Figure:**
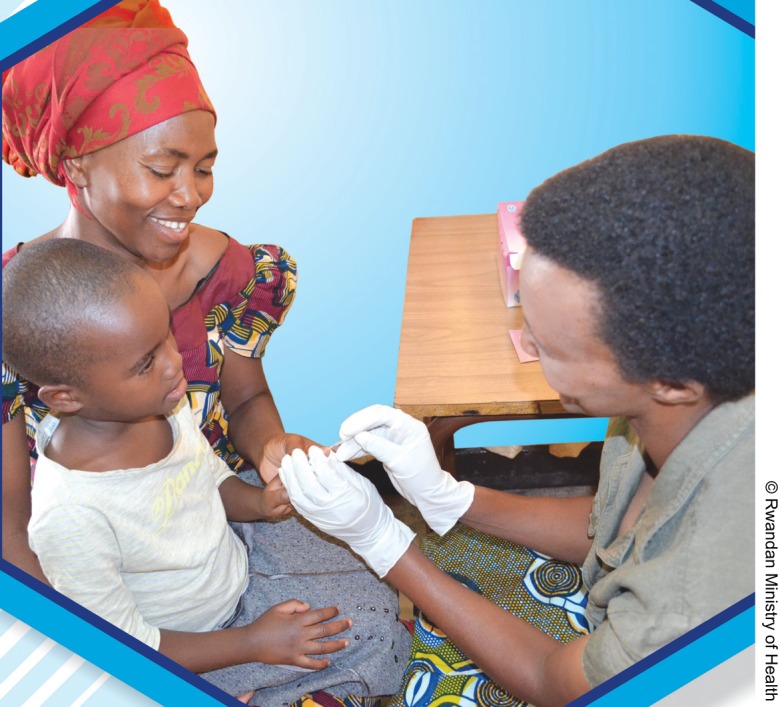
A community health worker draws blood from a child for malaria testing.

Total health facility use for children under 5 declined significantly after iCCM implementation.

### Comparison of Actual Versus Expected Trends

Using data from the CME Info database, we modeled an 11% annual reduction in total under-5 mortality for Rwanda between 1998 and 2008.^8^ Applying this estimated trend to our baseline pre-iCCM data in each district, we calculated an expected average decline of 0.05 deaths/1,000 child-months over the year following iCCM implementation (Table 3). We found that the actual decrease of 0.19 deaths/1,000 child-months was significantly greater than the expected decrease for the 15 districts in which complete data were available (*P* = .003).

**Table 3.** Comparison of Expected With Actual Declines in Child Mortality and Health Facility Use, Rwanda

**Indicator (No. of Districts)**	**Expected**	**Actual**	**Difference (95% CI)**	***P* Value (2-tailed)**
Health facility use (19)	0.05	1.04	1.00 (0.16, 1.84)	.03
Total mortality (15)	0.05	0.19	0.13 (0.06, 0.21)	.003

All data are reported as number per 1,000 child-months. Because to rounding, differences may not appear exact.

To compare actual versus expected trends in health facility use, we used HMIS data to calculate a baseline annual percentage change in health facility use for the 19 districts included in our analysis. Applying this annual percentage change in health facility use to the baseline pre-iCCM average for each district, we expected an average decline of 0.05 contacts/1,000 child-months in the year following iCCM implementation (Table 3). We found that the actual decrease of 1.04 contact/1,000 child-months was significantly greater than the expected decrease (*P* = .03).

## DISCUSSION

### Relationship to Prior Studies

While a few studies have examined the impact of iCCM on child mortality in individual districts of low-income countries, and others have looked at indirect measures of iCCM efficacy at the national level, to our knowledge, this is the first study to explore both changes in health facility use and in child mortality after iCCM implementation at the national level.^13^^–^^28^ The results of our study are, however, in line with others carried out in similar settings worldwide.

In Chokwe District, Mozambique, researchers observed a 62% decline in under-5 mortality with the introduction of a community-based child survival program.^18^ A 3-year study conducted in Gadchiroli District, India, demonstrated a 45.7% decrease in infant mortality in areas where home-based neonatal care was introduced compared with control areas.^29^ A randomized control trial of both facility- and community-based pediatric case management in the Matlab District, Bangladesh, found a non-significant trend toward decreased mortality in intervention areas.^13^

A few studies conducted at the national level have examined the impact of different types of community-based interventions on child health. In Nepal, between 2004 and 2007 a community-based program focused on acute respiratory infection and diarrhea case management was scaled up in 48 of the country's 75 districts. Using MOH data, researchers noted a decline in cases of severe pneumonia and dehydration, although they did not report a decline in total under-5 mortality.^20^ A descriptive study of the Lady Health Workers Program (LHWP) in Pakistan, a community-based program to improve maternal and child health covering an estimated 60% of the population nationally, found that infant mortality in 2007 was lower in LHWP areas compared with the national average, although the study did not directly compare intervention and non-intervention areas.^23^

Prior studies have also examined process measures of iCCM performance. Studies conducted in several developing countries have demonstrated high rates of appropriate management of pediatric pneumonia, diarrhea, and malaria by CHWs.^13^^,^^15^^,^^21^^,^^22^^,^^24^^,^^25^^,^^27^^,^^28^^,^^30^^–^^32^ In Rwanda, a 2010 rapid evaluation of the iCCM program in the 17 districts where CHWs had been practicing iCCM for at least 3 months concluded that 68% of children with pneumonia, 72% of children with diarrhea, and 86% of children with malaria were prescribed the appropriate course of treatment.^8^

Additional studies have demonstrated improvements in caregiver knowledge and immunization rates with implementation of iCCM programs.^13^^,^^17^^,^^23^^,^^33^ One study in Honduras documented a decrease in health facility use and significant cost savings with the implementation of a community case management program.^19^

### Relationship to DHS Data

The results of our study are also in line with those of the 2010 Rwanda DHS, although there are important differences in the methods used to collect the data in the DHS and HMIS databases. The 2010 DHS was conducted prior to complete implementation of iCCM nationwide in Rwanda, which was accomplished in 2011. Even so, the 2010 DHS found that 13%–16% of children in Rwanda experiencing symptoms of acute respiratory infection, diarrhea, and fever were seen and evaluated by a CHW.^34^ While this proportion might seem low, a recently published review found that Rwanda had the highest rates of CHW use in the region for both acute respiratory infections and diarrhea.^35^

It is important to note, however, that while the DHS measures the proportion of children with specific symptoms who were *evaluated* by a CHW in the 2 weeks prior to the survey, the Rwanda HMIS instead tracks the number of children who were *treated* by a CHW for specific symptoms each month. According to both international guidelines and the Rwanda trainer's guide for CHWs, only a fraction of children with cough, diarrhea, and fever require specific treatment; most simply require reassurance and clear instructions to the parent about when to return for worsening symptoms. Table 4 compares the DHS and HMIS data, providing a glimpse into the proportion of children with various symptoms evaluated by CHWs in Rwanda who received a specific treatment for those symptoms.

**Table 4.** Comparison of Rwanda 2010 DHS With 2010 HMIS Data for CHW Use

**Illness**	**Rwanda 2010 DHS**			**Rwanda 2010 HMIS**	**Comparison**
	**Proportion With Symptoms in Past 2 Weeks**	**Proportion Seeking Care or Advice From CHW**	**No. Children/1,000 Child-Months Seeking Care or Advice From CHW**	**No. Children/1,000 Child-Months Receiving Treatment From CHW**	**Proportion of Those Seeking Care Who Received Treatment From CHW**
ARI	4%	13%	9.62	5.28	55%
Diarrhea	13%	13%	34.06	3.80	11%
Fever	16%	16%	49.30	7.27	15%

Abbreviations: ARI, acute respiratory infection; CHW, community health worker; DHS, Demographic and Health Survey; HMIS, health management information system.

Comparison of the DHS and HMIS data also provides insight into the proportion of deaths being captured by the HMIS. While the DHS provides an unbiased estimate of the actual under-5 mortality rate (76 deaths per 1,000 live births in 2010), the HMIS data captures only those deaths that come to the attention of the CHW or that occur in the health center or hospital setting (0.49 deaths per 1,000 child-months); HMIS data, therefore, miss deaths occurring at home that are never reported.

In order to compare the DHS mortality data to the HMIS data, certain calculations must be conducted first since the DHS measures child mortality in terms of deaths per 1,000 live births and the HMIS measures child mortality in terms of monthly deaths per 1,000 children in the population. For the data to be comparable, we must first multiply the DHS figure of 76/1,000 by the Rwanda live birth rate (approximately 2.6%), and then divide by the proportion of the population under 5 (16.2%), and finally divide by 12 months per year. The result is 1.02 deaths per 1,000 child-months. The HMIS number of 0.49 per 1,000 child-month is 48% of the DHS number, suggesting that the HMIS database captures about half of all child deaths in Rwanda. In fact, the proportion is likely somewhat larger, since the mortality rate in the 2010 DHS is an average for the years 2006–2010; the actual mortality rate in 2010 is likely lower than 76, making the proportion of deaths captured by the HMIS database somewhat larger than 48%.

The Rwanda HMIS database captures about 48% of all under-5 child deaths.

### Relationship to Other Public Health Interventions

Although the declines in health facility use and all-cause under-5 mortality in the year after iCCM implementation are greater than would be expected due to baseline trends in Rwanda, these changes could be due in part to other major public health interventions introduced around the same time as iCCM in any given district, such as mass distribution of long-lasting insecticide-treated nets (LLINs) for malaria prevention; introduction of the pneumococcal conjugate vaccine (PCV) as part of the routine childhood vaccination schedule; and introduction of the rotavirus vaccine.

In September 2006, Rwanda instituted mass, nationwide distribution of LLINs for all children under 5, followed afterwards by routine distribution of LLINs to pregnant women at their second antenatal care visit and to children at their 9-month measles vaccination beginning in December 2006.^36^ Given that routine distribution of LLINs began almost 2 years prior to the introduction of iCCM in any district in Rwanda, it is unlikely to have affected the changes in mortality and health facility use between the baseline and comparison periods in our study.

PCV was introduced in April 2009 as part of the national routine childhood vaccination schedule; in relation to our study, this occurred between the baseline and comparison period for 13 districts and prior to the baseline period for 17 districts, including all 15 districts that were in our mortality analysis. Based on the best available data in the literature, PCV has been shown to decrease rates of all-cause child mortality by 11%,^37^ so it certainly may have contributed to a portion of the 38% decline in total all-cause under-5 mortality noted in our study.

Rotavirus immunization, on the other hand, was introduced into the routine childhood vaccination schedule in May 2012, which was after the comparison period for all districts in our study, and therefore could not have impacted our findings.

### Limitations

As described earlier, our study uses Rwanda HMIS data to estimate changes in under-5 mortality. Prior research has shown that vital registry data underestimate under-5 mortality compared with data obtained from household surveys,^18^ and in our study the HMIS data likely capture only about half of all child deaths. While national household-based birth history data are available from the 2010 Rwanda DHS, this survey provides only a 5-year average of national under-5 mortality, not district-level, monthly totals of under-5 deaths, which are needed to examine the impact of iCCM in the first year of implementation in each district.^38^ Although our community-based HMIS data likely underestimate under-5 deaths, we expect them to underestimate deaths similarly in both the baseline and implementation periods. While it is possible that the completeness of CHW data collection improved over time, this would mean that actual decreases in child mortality were even greater than our estimates suggest.

The HMIS data presented in this study provide the absolute number of children treated for pneumonia, diarrhea, and malaria by CHWs but do not describe the quality or appropriateness of that treatment. However, the proportion of children treated for acute respiratory infection (Table 4) is similar to the proportion of children with cough found to have pneumonia in prior facility-based research in Rwanda,^39^ and the proportion of children with fever treated with an antimalarial is also similar to the proportion of children with fever in Rwanda found to have malaria in prior research.^40^ While the rate of treatment with ORS and zinc for diarrhea seems low, this may be due to differences in the way diarrhea was assessed in the DHS (caregiver report of any diarrhea symptoms) compared with the way it was assessed by CHWs (at least 3 loose stools in a 24-hour period).

Data quality at the community level remains another limitation, partly due to limited CHW numeracy. A recent study of CHW reporting in one Rwandan district during May–June 2011 found that only 57% and 79% of monthly village CHW reports agreed perfectly with the tally of individual sick-child encounter forms for the number of children treated for pneumonia and malaria, respectively.^41^ However, as pointed out in the study, the quality of CHW data collection is likely to be better for more noteworthy events, such as child deaths, where the total number for a village in a given month is almost certain to be either 0 or 1, and would not at all impact the health facility use figures, which are based on actual visits to health centers and admissions to hospitals.

Missing data in the HMIS database is another limitation. To control for the effect of missing data, we performed a more conservative analysis that eliminates districts with any missing data in either the pre-iCCM baseline or post-iCCM comparison period. The results of the conservative analyses were similar to those of our primary analyses, with the caveat that we had slightly less power to detect differences. This conservative analysis cannot control, however, for the absence of baseline community mortality data for districts that implemented iCCM prior to 2010, when regular collection of community mortality data began nationwide. As such, changes in community mortality with implementation of iCCM cannot be generalized to the entire country, but rather only to the 15 districts that implemented iCCM in 2010 and 2011.

Finally, while we have endeavored to control for baseline trends in both child mortality and health facility use using the best available data, we cannot completely disentangle the effects of iCCM from those of other major public health interventions instituted in Rwanda at about the same time as iCCM. As discussed earlier, we believe it unlikely that either the LLIN distribution in 2006 or the introduction of rotavirus vaccine in 2012 would have impacted our results, as these interventions were well outside the time frame for iCCM implementation. However, it is certainly plausible that the introduction of PCV in 2009 may have contributed to a portion of the 38% decline in total all-cause under-5 mortality noted in our study.

## CONCLUSION

Between 2008 and 2011, Rwanda brought iCCM to scale in all 30 of its districts nationwide. We find significant increases in community-level treatment rates for childhood diarrhea and pneumonia during the 1-year period after iCCM implementation in each district. These increases correspond with decreases in under-5 community mortality, total mortality, health center use, and total health facility use. Moreover, the decreases in total under-5 mortality and health facility use are greater than would be expected due to baseline trends. Due to limitations in our study design, including the lack of a co-temporal control group for comparison, we cannot entirely attribute these decreases to the implementation of iCCM. In addition, each nation is unique and the experience of iCCM implementation in Rwanda cannot be directly extrapolated to other resource-limited settings, which may lack the highly organized and well-regulated government health system present in Rwanda. However, our study demonstrates that with sustained political will, reliable financial support, and robust monitoring and evaluation, community case management can be effectively brought to scale in a low-income country and, alongside other important public health interventions, may help reduce both health facility use and child mortality.

## Notes

**Peer Reviewed**

**First published online:** 2014 Aug 5
